# Limited generalizability of single deep neural network for surgical instrument segmentation in different surgical environments

**DOI:** 10.1038/s41598-022-16923-8

**Published:** 2022-07-22

**Authors:** Daichi Kitaguchi, Toru Fujino, Nobuyoshi Takeshita, Hiro Hasegawa, Kensaku Mori, Masaaki Ito

**Affiliations:** 1 Surgical Device Innovation Office, National Cancer Center Hospital East, 6-5-1, Kashiwanoha, Kashiwa, Chiba 277-8577 Japan; 2 Department of Colorectal Surgery, National Cancer Center Hospital East, 6-5-1, Kashiwanoha, Kashiwa, Chiba 277-8577 Japan; 3 Graduate School of Informatics, Nagoya University, Furo-cho, Chikusa-ku, Nagoya, Aichi 464-8601 Japan

**Keywords:** Colorectal cancer, Information storage

## Abstract

Clarifying the generalizability of deep-learning-based surgical-instrument segmentation networks in diverse surgical environments is important in recognizing the challenges of overfitting in surgical-device development. This study comprehensively evaluated deep neural network generalizability for surgical instrument segmentation using 5238 images randomly extracted from 128 intraoperative videos**.** The video dataset contained 112 laparoscopic colorectal resection, 5 laparoscopic distal gastrectomy, 5 laparoscopic cholecystectomy, and 6 laparoscopic partial hepatectomy cases. Deep-learning-based surgical-instrument segmentation was performed for test sets with (1) the same conditions as the training set; (2) the same recognition target surgical instrument and surgery type but different laparoscopic recording systems; (3) the same laparoscopic recording system and surgery type but slightly different recognition target laparoscopic surgical forceps; (4) the same laparoscopic recording system and recognition target surgical instrument but different surgery types. The mean average precision and mean intersection over union for test sets 1, 2, 3, and 4 were 0.941 and 0.887, 0.866 and 0.671, 0.772 and 0.676, and 0.588 and 0.395, respectively. Therefore, the recognition accuracy decreased even under slightly different conditions. The results of this study reveal the limited generalizability of deep neural networks in the field of surgical artificial intelligence and caution against deep-learning-based biased datasets and models.

**Trial Registration Number:** 2020-315, date of registration: October 5, 2020.

## Introduction

Minimally invasive surgery (MIS), including robotic surgery, has become increasingly common^1^. MIS that uses scopes to observe internal anatomy is preferred for many surgical procedures because a magnified surgical field of view can be obtained through the scope. Furthermore, surgical procedures can be stored as video data; therefore, this approach facilitates not only surgical training and education but also surgical data science^2^, such as computer vision using deep learning.

Computer vision is a research field that describes the machine understanding of images and videos, and significant advances have resulted in machines achieving human-level capabilities in areas such as object and scene recognition^3^. The main healthcare-related work in computer vision is computer-assisted diagnosis, such as colonic polyp detection^4,5^ and skin cancer detection^6,7^; however, the application of computer-assisted surgery has also accelerated^8,9^. In particular, surgical-instrument segmentation and the tracking of their tips are important underlying technologies because they can be applied to surgical skill assessment^10,11^, and they are essential for the achievement of automatic and autonomous surgery^12^.

Segmentation is a computer-vision task in which whole images are divided into pixel groups that can be labeled and classified. In particular, semantic segmentation attempts to semantically understand the role of each pixel in images^13^. Instance segmentation, which extends semantic segmentation, segments different instances of classes, i.e., labeling five individuals with five different colors; therefore, it can identify the boundaries, differences, and relations between objects for multiple overlapping objects^14^.

These computer-vision approaches have great applicability to surgical-instrument recognition in intraoperative videos for MIS, and, in recent years, there have been numerous efforts to develop surgical-instrument segmentation^15,16^. Among them, the Medical Image Computing and Computer Assisted Interventions Society has held international challenges based on recognition accuracy for surgical-instrument segmentation and the Endoscopic Vision Challenge^15,17–19^; novel deep neural networks have broken the record for state-of-the-art segmentation accuracy. However, these efforts have been performed on video datasets corresponding to the same type of surgery using a fixed type of surgical instrument and the same type of laparoscopic recording system, unlike real-world surgical settings. Practically, there are many different conditions in real-world surgical situations. For example, different types of laparoscopic recording systems and laparoscopic surgical instruments are used in different hospitals; in addition, surgical devices are upgraded, and their shapes slightly change every few years. When considering the general-purpose properties of a single surgical-instrument recognition network, it is also important to verify the applicability of the network to other types of surgery, i.e., to clarify the difference in the recognition accuracy when a recognition network that was developed based on the data of a certain type of surgery is applied to another type of surgery. Although such conditions related to recognition accuracy can clarify that constructing an intraoperative video dataset with diversity is important, no comprehensive study on the generalizability of a single surgical instrument recognition network has been reported. Therefore, the results of this study are important because they provide valuable information for future surgical development and implementation.

This study aimed to evaluate the generalizability of a single deep neural network for comprehensive surgical-instrument segmentation, thereby clarifying the difference in segmentation accuracy when a single network is applied to different situations, such as the type of laparoscopic recording system, recognition target surgical instrument, and surgery.

## Material and methods

### Study design

This research involved a retrospective experimental observational study using a five-institutional intraoperative video dataset. A total of 5238 images, which were randomly extracted from 128 intraoperative videos, were utilized. The image selection criteria were that the target surgical instrument must be clearly visible, and out-of-focus images and/or images with mist were excluded. The video dataset contained 112 laparoscopic colorectal resection (LCRR), 5 laparoscopic distal gastrectomy (LDG), 5 laparoscopic cholecystectomy (LC), and 6 laparoscopic partial hepatectomy (LPH) cases.

This study followed the Strengthening the Reporting of Observational Studies in Epidemiology (STROBE) reporting guidelines^20^. The protocol for this study was reviewed and approved by the Ethics Committee of National Cancer Center Hospital East, Chiba, Japan (Registration No.: 2020-315). Informed consent was obtained in the form of an opt-out on the study website, and data from those who rejected participation were excluded. The study conformed to the provisions of the Declaration of Helsinki established in 1964 (and revised in Brazil in 2013).

### Training and test sets

**Figure 1:**
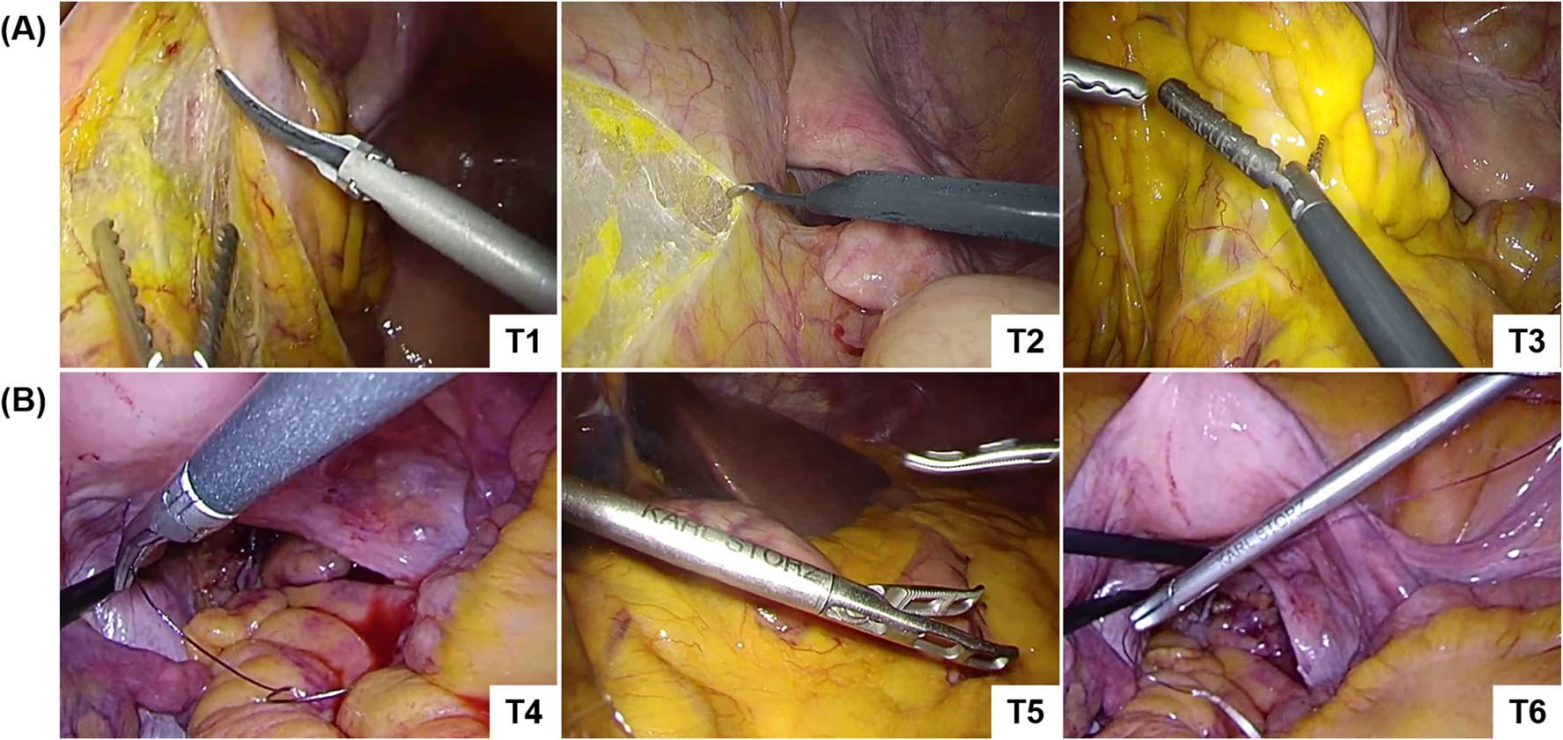
Representative images of recognition target surgical instruments in this study. (**A**) Surgical instruments contained in the training set (T1: harmonic shears; T2: endoscopic surgical electrocautery; T3: Aesculap AdTec atraumatic universal forceps). (**B**) Laparoscopic surgical forceps not contained in the training set (T4: Maryland; T5: Croce-Olmi; T6: needle holder).

The training set contained 4074 images, which were randomly extracted from 85 intraoperative videos of LCRR, and at least one of the following three types of surgical instruments was captured in each image: (T1) Harmonic Shears (Ethicon Inc., Somerville, NJ, USA), (T2) endoscopic surgical electrocautery (Olympus Co., Ltd., Tokyo, Japan), and (T3) Aesculap AdTec atraumatic universal forceps (B Braun AG, Melsungen, Germany). Representative images of T1–3 are shown in Fig. 1A. Every intraoperative video was recorded using an Endoeye laparoscope (Olympus Co., Ltd., Tokyo, Japan) and Visera Elite II system (Olympus Co., Ltd, Tokyo, Japan).

The validation set contained 345 images from nine intraoperative videos, and the conditions, which included the type of laparoscopic recording system, recognition target surgical instrument, and surgery, were the same as those for the training set.

Test set 1 contained 369 images from 10 intraoperative videos, and the conditions were the same as those of the training set.

Test set 2 contained 103 images, including surgical instruments extracted from five intraoperative videos. Although the recognition target surgical instrument and surgery types were the same as those in the training set, the videos were recorded using different types of laparoscopic systems, including a 1488 HD 3-Chip camera system (Stryker Corp., Kalamazoo, MI, USA) and Image 1 S camera system (Karl Storz SE & Co., KG, Tuttlingen, Germany).

Test set 3 contained 124 images that captured surgical instruments extracted from three intraoperative videos. Although the laparoscopic recording system and surgery types were the same as those of the training set, the types of recognition target were the following laparoscopic surgical forceps with slightly different tip shapes than T3: (T4) Maryland (Olympus Co., Ltd., Tokyo, Japan); (T5) Croce-Olmi (Karl Storz SE & Co., KG, Tuttlingen, Germany); (T6) needle holder (Karl Storz SE & Co., KG, Tuttlingen, Germany). T4–T6 were not included in the training set, and we tested whether they could be recognized as T3. Representative images of T4–T6 are shown in Fig. 1B.

Test set 4 contained 223 images that captured surgical instruments extracted from 16 intraoperative videos of different types of surgery, including LDG, LC, and LPH. The other conditions, including the types of laparoscopic recording system and recognition target surgical instrument, were the same as those for the training set.

Every image included in every set for training, validation, and test captured at least one type of surgical instrument. The characteristics of the training set, validation set, and each test set are summarized in Table 1.

**Table 1:** Dataset characteristics.

	Number of videos	Number of annotated images	Laparoscopic recording system	Recognition target surgical instruments	Type of surgery
Training set	85	4788	Olympus	T1, T2, T3	LCRR
Validation set	9	345			
Test set 1	10	369			
**Test set 2**	5	103		T1, T2, T3	LCRR
Sub test set 2.1	2	40	Stryker		
Sub test set 2.2	3	63	Karl Storz		
**Test set 3**	3	124			LCRR
Sub test set 3.1	1	31	Olympus	T4	
Sub test set 3.2	1	74		T5	
Sub test set 3.3	1	19		T6	
**Test set 4**	16	223		T1, T2, T3	
Sub test set 4.1	5	65	Olympus		LDG
Sub test set 4.2	5	81			LC
Sub test set 4.3	6	77			LPH

*T1* harmonic shears, *T2* endoscopic surgical electrocautery, *T3* Aesculap AdTec atraumatic universal forceps, *T4* Maryland, *T5* Croce-Olmi, *T6* needle holder; *LCRR* laparoscopic colorectal resection, *LDG* laparoscopic distal gastrectomy, *LC* laparoscopic cholecystectomy, *LPH* laparoscopic partial hepatectomy.

### Annotation

Annotation was performed by 14 nonphysicians under the supervision of surgeons, and all the annotated images were double-checked by surgeons. The annotation labels were manually assigned pixel by pixel by drawing directly on the area of each surgical instrument in the images using Wacom Cintiq Pro (Wacom Co., Ltd., Saitama, Japan) and Wacom Pro Pen 2 (Wacom Co., Ltd., Saitama, Japan). The representative annotated images are shown in Supplementary Fig. 1.

### Data pre-processing

Every intraoperative video was converted into MP4 video format with a display resolution of 1280 × 720 pixels and frame rate of 30 frames per second (fps), and neither upsampling nor downsampling was performed.

The data split was performed on the per-case level instead of the per-frame level; thus, no image extracted from an intraoperative video in the training set appeared in the test sets.

### Model optimization

A mask region-based convolutional neural network (R-CNN) with a deformable convolution^14,21^ and ResNet50^22^ were utilized as the instance-segmentation model and backbone network, respectively, and every annotated image in the training set was input into the model. The model architecture and workflow of the deep neural network are shown in Supplementary Fig. 2. The network weight was initialized to a pre-trained one on the ImageNet^23^ and COCO^24^ datasets, and fine-tuning was then performed for the training set. ImageNet is a large visual database designed for use in visual object recognition tasks. It contains more than 14 million images with labels of more than 20,000 typical categories, such as “balloon” and “strawberry.” COCO is a large-scale dataset for object detection, segmentation, and captioning. It contains more than 120,000 images with more than 880,000 labeled instances for 80 object types.

The model was trained and tested to distinguish between T1, T2, and T3. For test set 3, the model was tested for if T4, T5, and T6 could be recognized as T3. The best epoch model based on the model performance on the validation set was selected. Horizontal and vertical flips were used for data augmentation. The hyperparameters used for the model training are listed in Supplementary Table 1.

### Code and computer specification

The code was written using Python 3.6 (Python Software Foundation, Wilmington, DE, USA), and the model was implemented based on MMDetection^25^, which is an open-source Python library for object detection and instance segmentation.

A computer equipped with an NVIDIA Quadro GP100 GPU with 16 GB of VRAM (NVIDIA, Santa Clara, CA, USA) and Intel^®^ Xeon^®^ CPU E5-1620 v4 @ 3.50 GHz with 32 GB of RAM was utilized for network training.

### Model performance

The intersection over union (IoU) and average precision (AP) were utilized as metrics to assess the model performance for the surgical-instrument-segmentation task.

The IoU was calculated for each pair of X (the area annotated as the ground truth) and Y (predicted area output by the model), which simply measures the overlap of the two areas divided by their union, as follows:

$$IoU = | X \cap Y | / | X \cup Y |.$$

The mean AP (mAP) is a metric that is widely used for object-detection and instance-segmentation tasks^23,24,26^. It is calculated from the area under the precision–recall curve that is described based on the number of true positives (TP), false negatives (FN), and false positives (FP). Assigned pairs of X and Y were defined as TP and FN when their IoU was more and less than 0.75, respectively, and they were defined as FP when no pairs could be assigned.

To confirm the reproducibility of the results, we trained five models for each test set with different random seeds and reported the metrics averaged over the five models as the mean (± standard deviation).

### Ethical approval

Ethics Committee of National Cancer Center Hospital East, Chiba, Japan (Registration No.: 2020-315).

### Informed consent

Informed consent was obtained in the form of an opt-out on the study website.

### Consent for publication

The authors affirm that the human research participants provided informed consent for the publication of the images in the figures.

## Results

**Figure 2:**
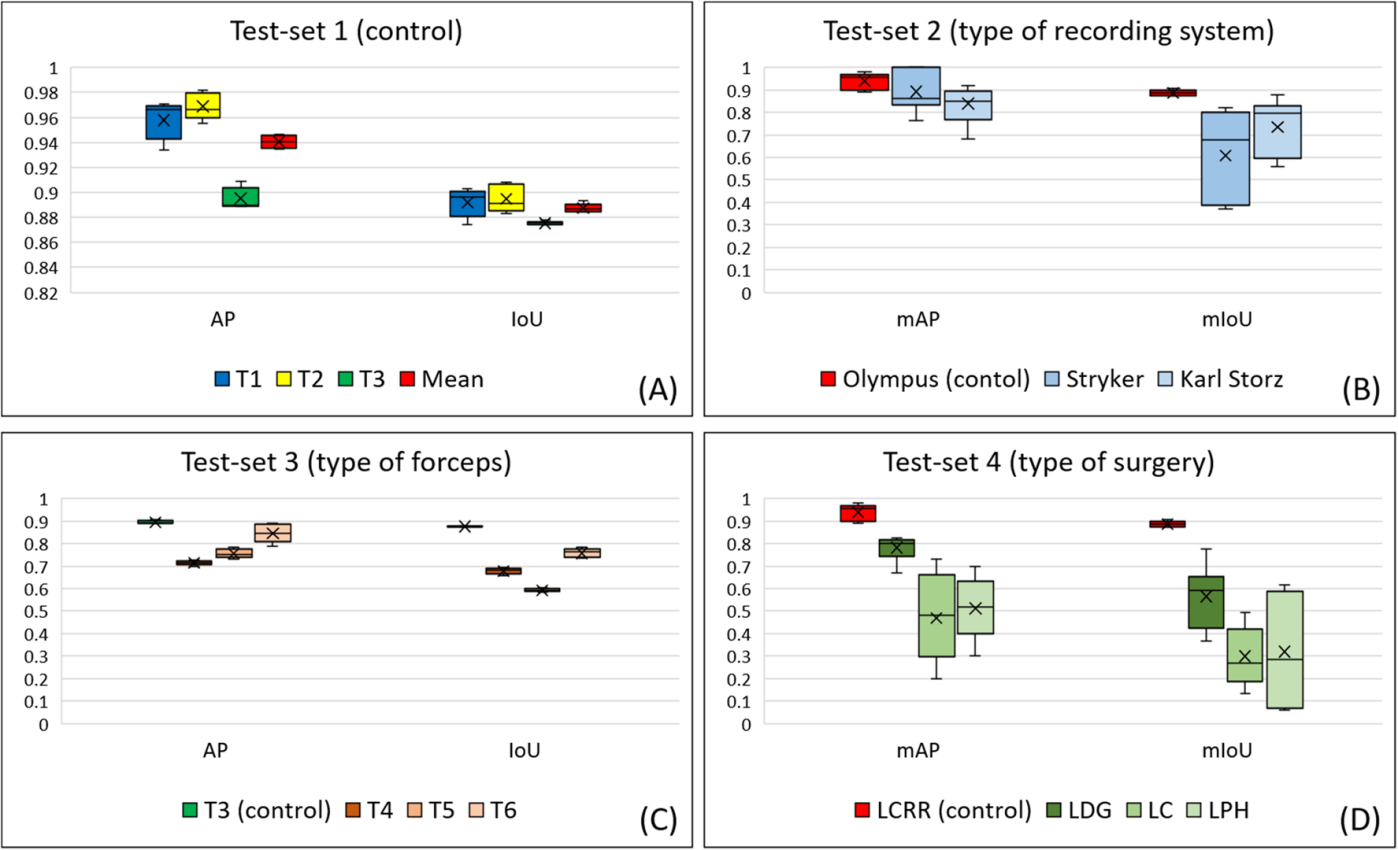
Surgical-instrument recognition-accuracy results (*AP* average precision, *IoU* intersection over union, *mAP* mean average precision, *mIoU* mean intersection over union). (**A**) AP and IoU under the same condition as the training set (T1: harmonic shears; T2: endoscopic surgical electrocautery; T3: Aesculap AdTec atraumatic universal forceps). (**B**) mAP and mIoU for different types of laparoscopic recording systems. (**C**) AP and IoU for different types of laparoscopic surgical forceps (T3: Aesculap AdTec atraumatic universal forceps; T4: Maryland; T5: Croce-Olmi; T6: needle holder). (**D**) mAP and mIoU for different types of surgery (*LCRR* laparoscopic colorectal resection, *LDG* laparoscopic distal gastrectomy, *LC* laparoscopic cholecystectomy, *LPH* laparoscopic partial hepatectomy).

The results for test set 1 are shown in Fig. 2A. The mAP and mean IoU (mIoU) for test set 1 were 0.941 (± 0.035) and 0.887 (± 0.012), respectively, and the AP and IoU for T1, T2, and T3 were 0.958 and 0.892, 0.969 and 0.895, and 0.895 and 0.876, respectively (Fig. 2A). These results were utilized as control values for comparison in this study.

**Figure 3:**
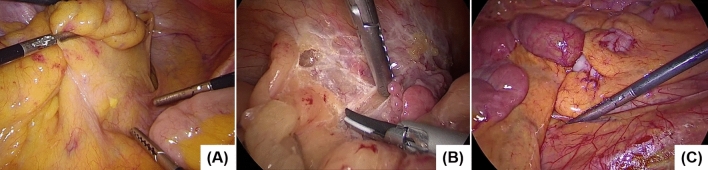
Representative images recorded by each laparoscopic recording system. (**A**) Endoeye laparoscope (Olympus Co., Ltd., Tokyo, Japan) and Visera Elite II system (Olympus Co., Ltd, Tokyo, Japan). (**B**) 1488 HD 3-Chip camera system (Stryker Corp., Kalamazoo, MI, USA). (**C**) Image 1 S camera system (Karl Storz SE & Co., KG, Tuttlingen, Germany).

The mAP and mIoU for test set 2 were 0.866 (± 0.035) and 0.671 (± 0.082), respectively. These results indicate that when different laparoscopic recording systems were utilized, the mAP and mIoU slightly deteriorated as compared with the control values, even though the other conditions were the same as for the training set. The mIAP and mIoU values that were acquired when using the laparoscopic recording systems produced by the Stryker and Karl Storz cameras were 0.893 and 0.608 and 0.839 and 0.735, respectively (Fig. 2B). The representative images recorded by each laparoscopic recording system are shown in Fig. 3. Each color tone is slightly different, even in the macroscopic observation.

The mAP and mIoU for test set 3 were 0.772 (± 0.062) and 0.676 (± 0.072), respectively. Although T4–T6 are also classified as laparoscopic surgical forceps in a broad sense, the recognition accuracy for T4–T6 deteriorated as compared with that for T3. The AP and IoU for T4, T5, and T6 were 0.715 and 0.678, 0.756 and 0.592, and 0.846 and 0.758, respectively (Fig. 2C).

**Figure 4:**
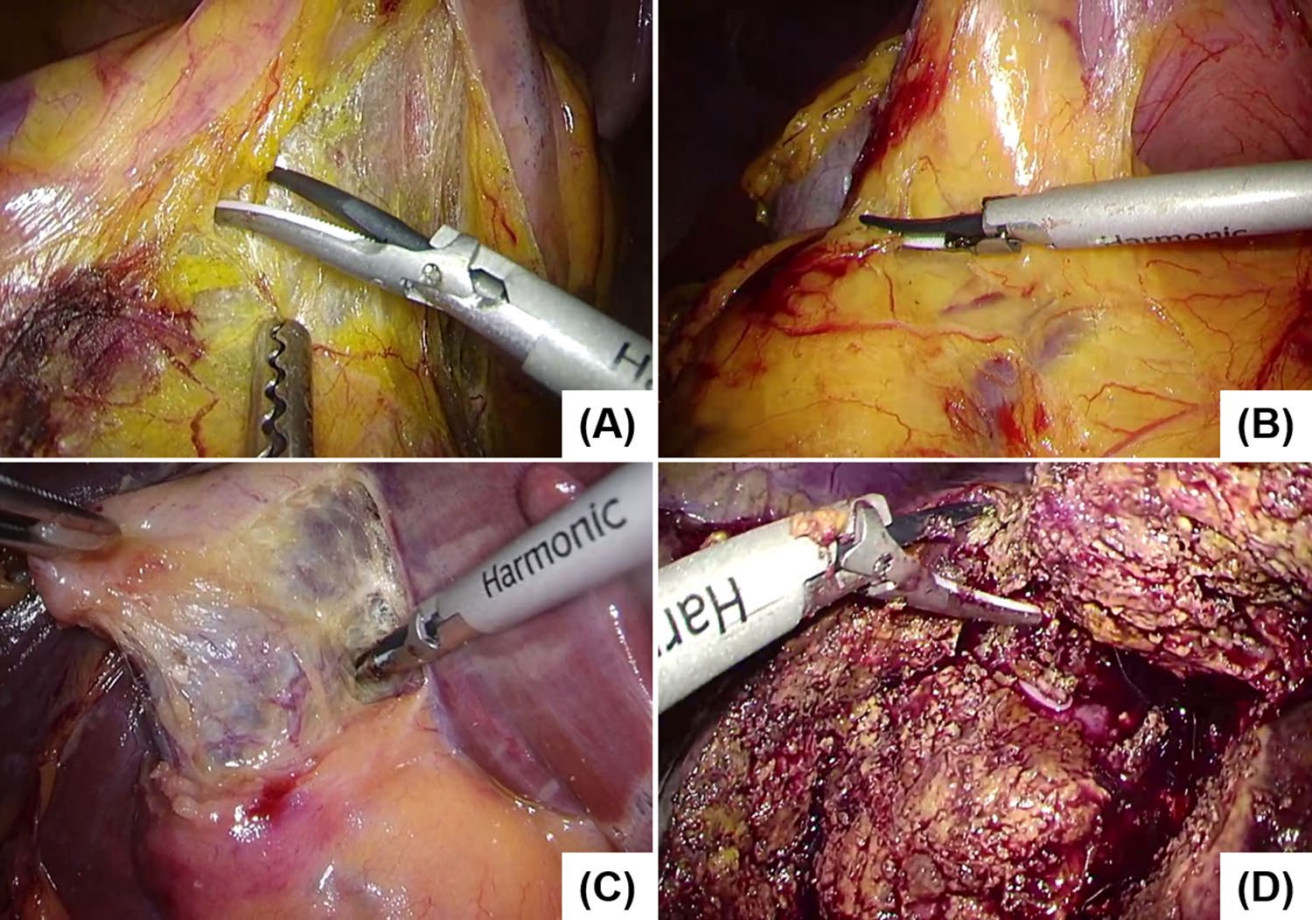
Representative images of each type of surgery. (**A**) LCRR; (**B**) LDG; (**C**) LC; (**D**) LPH.

The mAP and mIoU for test set 4 were 0.588 (± 0.151) and 0.395 (± 0.127), respectively. For a different type of surgery, the mAP and mIoU values significantly deteriorated as compared with the control values, even though the other conditions were the same as for the training set. The mAP and mIoU for LDG, LC, and LPH were 0.782 and 0.565, 0.468 and 0.300, and 0.513 and 0.319, respectively (Fig. 2D). The representative images for each type of surgery are shown in Fig. 4. The foreground surgical instruments are the same, especially in LC and LPH; however, the background is significantly different from the LCRR case, even for the macroscopic observation.

The surgical-instrument segmentation accuracy and representative segmentation results for each test set are shown in Table 2 and Supplementary Fig. 3, respectively.

**Table 2:** Surgical-instrument segmentation accuracy for each test set.

	AP	IoU
**Test set 1**		
T1	0.958 (± 0.015)	0.892 (± 0.011)
T2	0.969 (± 0.011)	0.895 (± 0.011)
T3	0.895 (± 0.009)	0.876 (± 0.001)
Mean	0.941 (± 0.035)	0.887 (± 0.012)
**Test set 2**		
Sub test set 2.1 (Stryker)	0.893 (± 0.021)	0.608 (± 0.068)
Sub test set 2.2 (Karl Storz)	0.839 (± 0.021	0.735 (± 0.019)
**Test set 3**		
Sub test set 3.1 (T4)	0.715 (± 0.010)	0.678 (± 0.014)
Sub test set 3.2 (T5)	0.756 (± 0.020)	0.592 (± 0.008)
Sub test set 3.3 (T6)	0.846 (± 0.041)	0.758 (± 0.020)
**Test set 4**		
Sub test set 4.1 (LDG)	0.782 (± 0.013)	0.565 (± 0.025)
Sub test set 4.2 (LC)	0468 (± 0.071)	0.300 (± 0.022)
Sub test set 4.3 (LPH)	0.513 (± 0.051)	0.319 (± 0.022)

Mean (± SD).

*AP*: average precision, *IoU* intersection over union, *T1* harmonic shears, *T2* endoscopic surgical electrocautery, *T3* Aesculap AdTec atraumatic universal forceps, *T4* Maryland, *T5* Croce-Olmi, *T6* needle holder, *LDG* laparoscopic distal gastrectomy, *LC* laparoscopic cholecystectomy, *LPH* laparoscopic partial hepatectomy, *SD* standard deviation.

## Discussion

In this study, we demonstrated that our surgical-instrument-segmentation network possesses high accuracy (mAP: 0.941, mIoU: 0.887). However, the generalizability of a single deep neural network applied to laparoscopic surgery has limitations, i.e., a minor change in the laparoscopic surgery conditions significantly affects the recognition accuracy of the surgical instrument.

First, these results suggest that the intraoperative video dataset recorded by a single laparoscopic recording system is insufficient to generalize a deep neural network. The recognition accuracy for test set 2 slightly deteriorated because the color tone was slightly different between the images recorded by each system even though the same objects were captured in each image. Second, because there are numerous types of surgical instruments, differences between hospitals, and updates to the versions of surgical devices produced by each company every several years, the training set needs to be updated as the device lineups and versions at the hospitals change. Third, even if a highly accurate surgical-instrument recognition network is successfully developed for one type of surgery, it cannot be applied to other types of surgery with similar accuracy. In particular, the more different the image background from the training set, the lower the recognition accuracy. In summary, diversity in the training set in terms of the type of laparoscopic recording system, types and versions of surgical instruments, and type of surgery used as the image background are considered crucial when applying a deep neural network to multi-institutional surgery in a real-world surgical setting.

Several previous scholars have investigated the generalizability of deep neural networks, specifically, the so-called “domain shift”, which refers to the training of a network on data from one domain and applying it to data from another. Zech et al. investigated the training of a CNN for pneumonia screening on chest X-rays generalized to new cohorts, and they identified significantly lower performance when the network was applied to X-ray images collected from hospitals that were not included in the training set^27^. Previous researchers have investigated CNN-based brain magnetic resonance imaging (MRI) image recognition performance and demonstrated that the performance of a CNN trained on MRI images from homogeneous research cohorts generally decreases when it is applied to other cohorts^28,29^. However, to the best of our knowledge, the present study is the first in which the generalizability of a single deep neural network for surgical instrument segmentation has been comprehensively investigated.

Automatic surgical-instrument recognition can be applied to the following two major research fields: robotics and skill assessment. Visual servoing is “actively controlled”, which means that it uses visual information to control the pose of the robot end effector relative to a target object^30^. Laparoscope-holder robots with visual servoing may assist surgeons in fully concentrating on the surgical task. In laparoscope-holder robots, the key to visual servoing is the marker-free tracking framework of the surgical instruments^31,32^. Therefore, in the future of the surgical field, automatic surgical-instrument recognition technology will play a pivotal role in the development of laparoscope-holder robots and the realization of autonomous MIS. Surgical skill assessment tools, such as the Objective Structured Assessment of Technical Skills^33^ and the Global Operative Assessment of Laparoscopic Skills^34^, have been utilized to objectively evaluate the basic surgical skills of surgical trainees; however, these tools rely on the observations and judgments of an individual^35^, which are inevitably associated with subjectivity and bias. Therefore, fair and objective automatic surgical skill assessment without a time-consuming video-review process has attracted attention in recent years. Automatic surgical-instrument recognition also plays a pivotal role in extracting kinematic data associated with surgical skills in MIS.

In supervised deep-learning research, the expense and time consumption of the manual annotation process used to construct large-scale datasets that are representative of real-world settings are major limitations. Moreover, even if a deep neural network that can demonstrate high performance under specific conditions is developed for a surgical-instrument-segmentation task, its usefulness is limited because real conditions are diverse and variable, and it is almost impossible to consider all of them. Therefore, clarifying the conditions to which a single surgical-instrument segmentation network can be applied is highly important for future development and implementation in terms of reducing annotation cost and time. Because the results of this study demonstrated that even slight changes in the image background affect the surgical-instrument recognition accuracy, the omission of the annotation step is not recommended. Considering the characteristics of deep neural networks, especially CNN-based image recognition approaches wherein the extraction of features from every pixel in an image is attempted, these results appear reasonable. However, it might be possible to eliminate the man-hours required for annotation by introducing a semi-supervised segmentation network even in different surgical environments, and this should be verified in future studies.

There are several limitations to this study. First, the objective of this study was to clarify how the generalizability of deep neural networks was limited in the surgical artificial intelligence research field, and the caution against biased datasets and models based on them was also implied. The generalizability may be improved by introducing different data-augmentation methods or different model architectures; however, because it was not the primary objective of this study, it was not considered. Second, although the video dataset utilized in this study contained relatively large multi-institutional data, it was a retrospective experimental observational study, and prospective validation was not performed. Further, because the dataset contained only images with surgical instruments, the FP in images without surgical instruments was not reflected in the results. Third, although the study results are considered to be highly important benchmarks for future research and development using deep neural networks in surgery, they provide no direct clinical benefit at the moment because we are still in the initial phase.

In conclusion, in a surgical-instrument segmentation task, the generalizability of a single deep neural network is limited, i.e., the recognition accuracy deteriorates even under slightly different conditions. Consequently, to enhance the generalization ability of a deep neural network, it is crucial to construct a training set that considers the diversity of the surgical environment in a real-world surgical setting.

## Supplementary Information

Supplementary Information 1.

Supplementary Information 2.

Supplementary Information 3.

Supplementary Information 4.

## Data Availability

The datasets generated and analyzed during the present study are available from the corresponding author upon reasonable request.

## Code availability

Available via GitHub upon reasonable request.
