# Supplementary material for: Limited generalizability of single deep neural network for surgical instrument segmentation in different surgical environments
Source: Sci Rep. 2022 Jul 22;12:12575. doi: 10.1038/s41598-022-16923-8 (PMC9307578; doi:10.1038/s41598-022-16923-8)
Supplement: Supplementary file 1 — Supplementary Information 1. [file 41598_2022_16923_MOESM1_ESM.docx]

**Supplementary Information**

**Supplementary Table 1:** Hyperparameters for model training

| Epoch | 12 |
| --- | --- |
| Batch Size | 2 |
| Learning Rate | 0.0025 |
| Loss function (LF)  LF for Classification  LF for Detection | Cross-entropy loss  L1 loss |
| Optimizer | Stochastic gradient descent |

**Supplementary Figure 1** Representative annotated images. (A) Raw images; (B) annotation labels; (C) overlay images.

**Supplementary Figure 2** Architecture of model and workflow of deep neural network. (CNN: convolutional neural network; RPN: region proposal network; RoI: region of interest; FC: fully connected; Conv.: convolutional).

**Supplementary Figure 3** Representative segmentation results for each test set. (A) Surgical-instrument-segmentation results, which are relatively good, even in images recorded using other laparoscopic systems. (B) Misrecognition of surgical forceps as T1 instead of T3. (C) Misrecognition of spleen as T2 in the LDG, and instability of the surgical tool recognition accuracy in LC and LPH. (T1: harmonic shears; T2: endoscopic surgical electrocautery; T3: Aesculap AdTec atraumatic universal forceps; T4: Maryland; T5: Croce-Olmi; T6: needle holder; LDG: laparoscopic distal gastrectomy; LC: laparoscopic cholecystectomy; LPH: laparoscopic partial hepatectomy; PD: T1; LD: T2; Grasper: T3).
