# Supplementary figures and images for: Limited generalizability of single deep neural network for surgical instrument segmentation in different surgical environments
Source: Sci Rep. 2022 Jul 22;12:12575. doi: 10.1038/s41598-022-16923-8 (PMC9307578; doi:10.1038/s41598-022-16923-8)

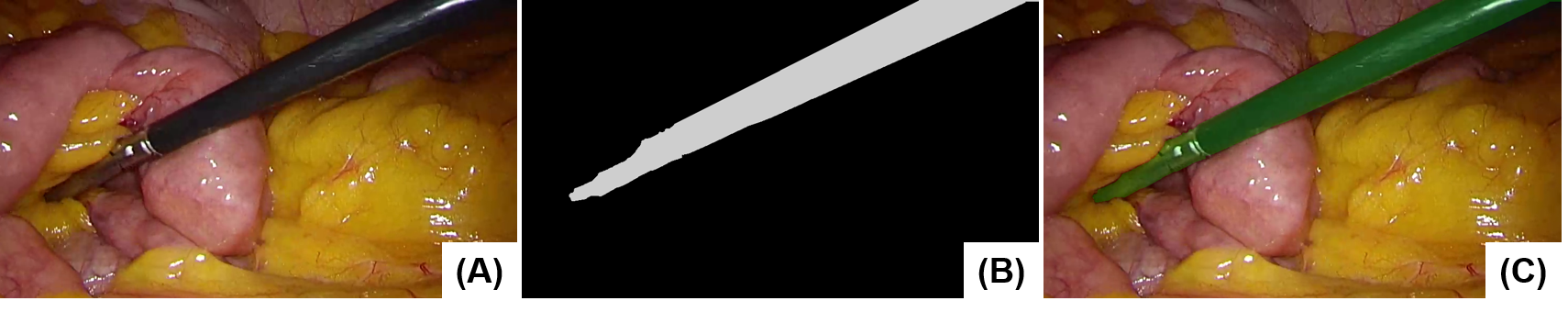

Supplement: Supplementary file 2 — Supplementary Information 2. [file 41598_2022_16923_MOESM2_ESM.tif]

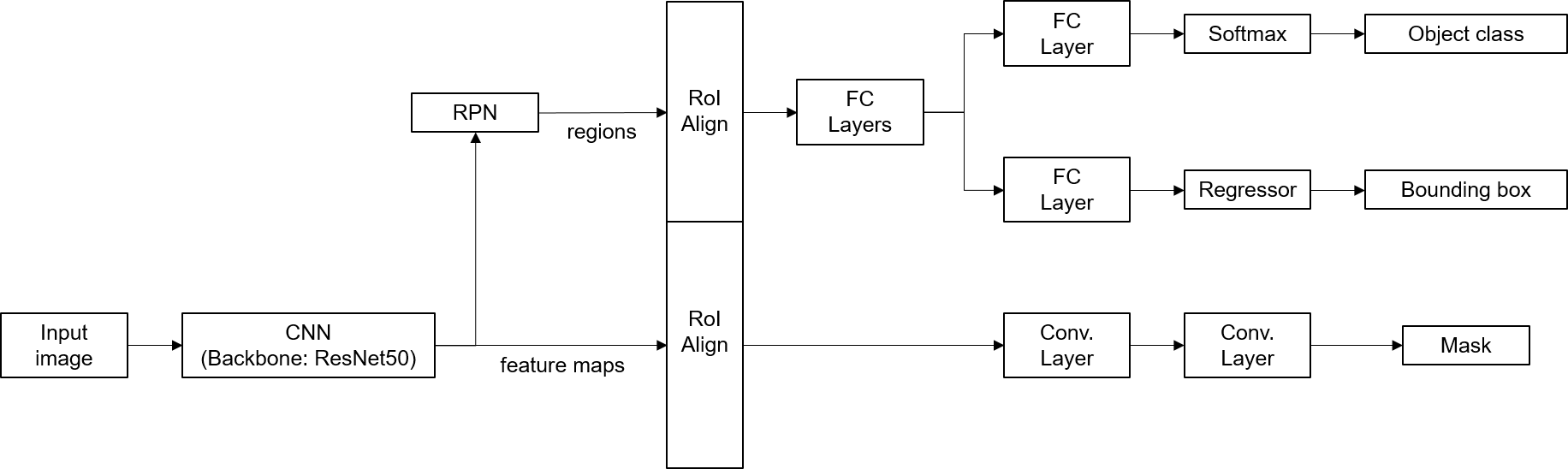

Supplement: Supplementary file 3 — Supplementary Information 3. [file 41598_2022_16923_MOESM3_ESM.tif]

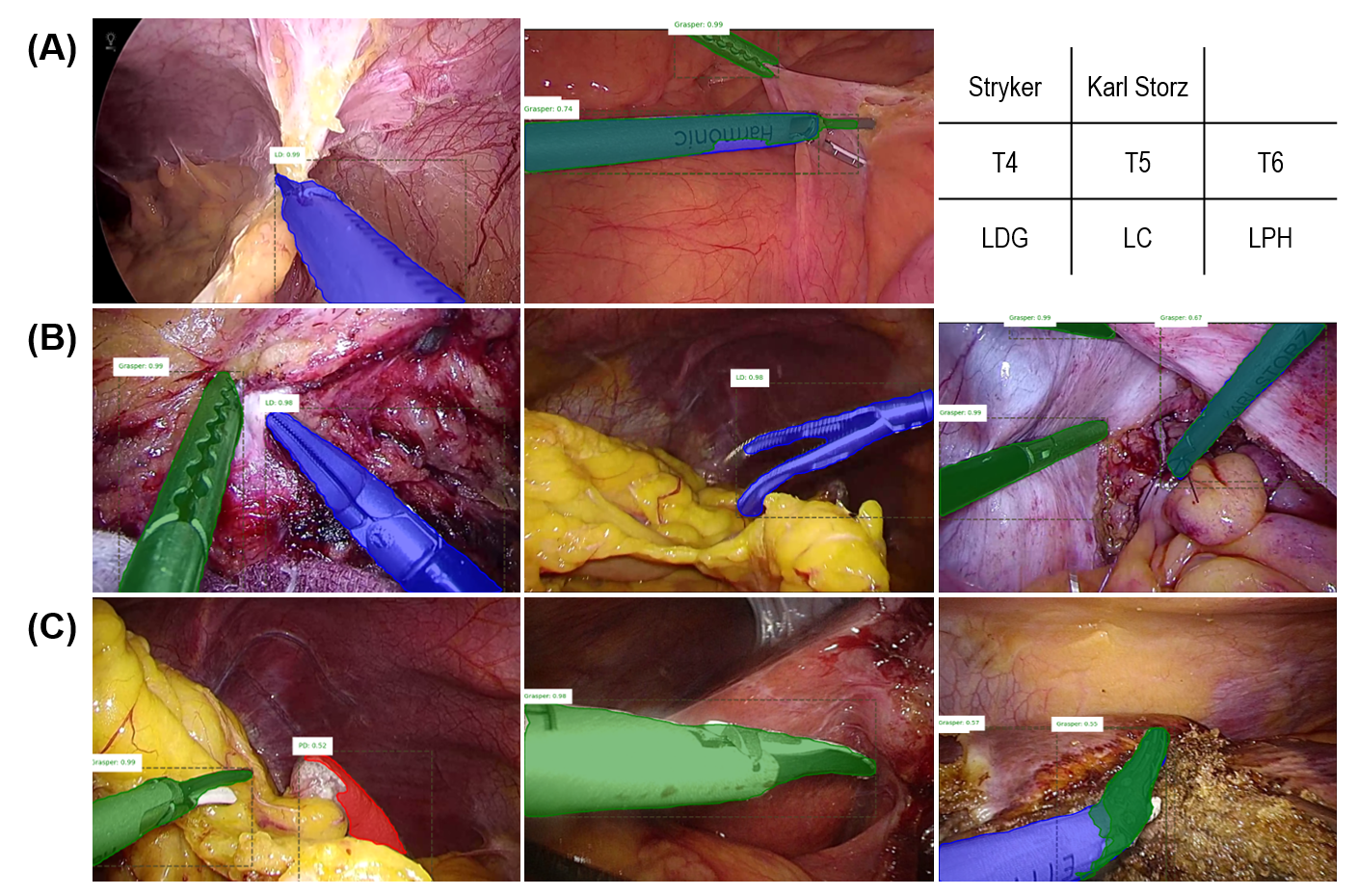

Supplement: Supplementary file 4 — Supplementary Information 4. [file 41598_2022_16923_MOESM4_ESM.tif]
